# Amelotin Gene Structure and Expression during Enamel Formation in the Opossum *Monodelphis domestica*

**DOI:** 10.1371/journal.pone.0133314

**Published:** 2015-07-17

**Authors:** Barbara Gasse, Xi Liu, Erwan Corre, Jean-Yves Sire

**Affiliations:** 1 Sorbonne Universités, UPMC Univ Paris 06, Institut de Biologie Paris-Seine (IBPS), UMR7138, 75005 Paris, France; 2 CNRS, IBPS, UMR7138, 75005 Paris, France; 3 Sorbonne Universités, UPMC Univ Paris 06, Station Biologique de Roscoff, Plateforme ABiMS (Analyses and Bioinformatics for Marine Science), 29680 Roscoff, France; University of Macau, MACAO

## Abstract

Amelotin (AMTN) is an ameloblast-secreted protein that belongs to the secretory calcium-binding phosphoprotein family, which also includes the enamel matrix proteins amelogenin, ameloblastin and enamelin. Although AMTN is supposed to play an important role in enamel formation, data were long limited to the rodents, in which it is expressed during the maturation stage. Recent comparative studies in sauropsids and amphibians revealed that (i) *AMTN* was expressed earlier, i.e. as soon as ameloblasts are depositing the enamel matrix, and (ii) *AMTN* structure was different, a change which mostly resulted from an intraexonic splicing in the large exon 8 of an ancestral mammal. The present study was performed to know whether the differences in *AMTN* structure and expression in rodents compared to non-mammalian tetrapods dated back to an early ancestral mammal or were acquired later in mammalian evolution. We sequenced, assembled and screened the jaw transcriptome of a neonate opossum *Monodelphis domestica*, a marsupial. We found two AMTN transcripts. Variant 1, representing 70.8% of AMTN transcripts, displayed the structure known in rodents, whereas variant 2 (29.2%) exhibited the nonmammalian tetrapod structure. Then, we studied AMTN expression during amelogenesis in a neonate specimen. We obtained similar data as those reported in rodents. These findings indicate that more than 180 million years ago, before the divergence of marsupials and placentals, changes occurred in *AMTN* function and structure. The spatiotemporal expression was delayed to the maturation stage of amelogenesis and the intraexonic splicing gave rise to isoform 1, encoded by variant 1 and lacking the RGD motif. The ancestral isoform 2, housing the RGD, was initially conserved, as demonstrated here in a marsupial, then secondarily lost in the placental lineages. These findings bring new elements towards our understanding of the non-prismatic to prismatic enamel transition that occurred at the onset of mammals.

## Introduction

Amelotin (AMTN) belongs to the secretory calcium-binding phosphoprotein (SCPP) family, and more precisely to the Pro-Glu rich sub-family, which includes the three enamel matrix proteins (EMPs), amelogenin (AMEL), ameloblastin (AMBN) and enamelin (ENAM) [1]. EMPs play an important role during enamel matrix formation, mineralization and maturation. The four genes have been found in tetrapod and coelacanth genomes [2] indicating a possible origin in early osteichthyans. However, only a few data are available on *AMTN* expression and on the supposed function(s) of the encoded protein. In mammals, our knowledge on *AMTN* expression was long limited to rodents [3], until our recent studies in a lizard and a salamander [4]. Comparison of *AMTN* expression revealed important spatiotemporal differences in rodents versus non-mammalian tetrapods. During rodent amelogenesis, *AMTN* is expressed by ameloblasts from maturation stage onwards [3] while in lizard and salamander, *AMTN* transcripts were detected from early enamel matrix formation to late enamel maturation [4]. These findings suggest different functions of AMTN during amelogenesis in non-mammalian tetrapods compared to rodents. In the latter it was suggested that AMTN could play a role in the adherence of ameloblasts and of the junctional epithelium to the enamel surface, or/and in the mineralization of the outer aprismatic enamel layer [5–8]. Data in non-mammalian tetrapods clearly indicate that the ancestral AMTN was an EMP and that some functional motifs were lost during mammalian evolution [4]. Evolutionary analyses in mammals, based on a large number of genomic sequences (gDNA), suggested that these changes occurred early in the mammalian lineage and that the origin of the current *AMTN* structure took place in the last common mammalian ancestor, i.e. more than 200 million years ago (Ma) [9]. The AMTN function(s) as proposed in rodents could date back to this ancient geological period (310–200 Ma), during which early mammals acquired dental occlusion and prismatic enamel [10].

The hypothesis that structural/functional changes in *AMTN* have occurred prior to the differentiation of the current mammalian lineages was recently questioned by Kawasaki and Amemiya [2]. In the opossum *Monodelphis domestica* (Marsupialia), these authors suggested that the *AMTN* structure was similar to that in non-mammalian tetrapods, i.e. a sequence ending with a large exon 8 encoding a RGD motif. This interpretation, also based on gDNA, implies that changes, which led to the current mammalian *AMTN* structure, i.e. a short exon 8 that does not encode the RGD followed with exon 9, occurred later in mammalian evolution. However, the only available cDNA data in rodents were not appropriate to accurately identify when this event took place during mammalian evolution. Moreover, in opossum *AMTN*, Kawasaki and Amemiya [2] identified a new putative exon between exons 2 and 3, absent in rodent cDNA. We identified this exon 2c in the cDNA sequences of non-mammalian tetrapods, and also suggested that this exon was present and functional in marsupial *AMTN* [4].

Accurate data based on cDNA sequences of *M*. *domestica AMTN* being necessary to confirm or invalidate these hypotheses, we undertook the present study to (i) define the correct *AMTN* structure and protein composition and (ii) analyze the spatiotemporal expression of *AMTN* during amelogenesis. Fulfilling these objectives was crucial to know when changes in structure and spatiotemporal expression took place in *AMTN* during mammalian evolution, and would bring new elements to our understanding of the non-prismatic to prismatic transition that occurs in enamel at the onset of mammals.

## Materials and Methods

The heads of two *Monodelphis domestica* neonates (16 days post fertilization), in which teeth are forming, were used in our study. The material was a gift from the King's College London facility lab (courtesy Dr Abigail Tucker). One head was preserved in RNALater for RNA extraction and sequencing, the other was fixed in PFA 4% and demineralized in EDTA 5% for *AMTN* expression study.

### RNA extraction and jaw transcriptome sequencing

The lower jaw was cut into small pieces and frozen in liquid nitrogen. Total RNA was extracted and aliquoted (detailed description in [4]). Illumina sequencing [one run, 50 base pair (bp) paired end] was commissioned to GATC Biotech. The sequenced transcriptome was assembled at ISEM-Montpellier 2 (France) using Trinity (release Apr13, 2014) the genome-independent transcriptome assembler using the default parameters [11]. Then the 192,056 sequences were screened for *AMTN* transcripts with BLAST on the Montpellier Bioinformatics Biodiversity (MBB) platform at [http://mbb.univ-montp2.fr/MBB/html_pise/BlastDB.html].

### PCR analysis and probe synthesis

Primers were designed with Primer3 v.0.4.0 (http://bioinfo.ut.ee/primer3-0.4.0/primer3/; [12]) using the two *AMTN* sequences obtained in the transcriptome. Two sets of primers were designed to obtain the 3' end of the transcripts, depending on the presence of either a large exon 8 alone (913 bp, Reverse 1) or a short exon 8 followed with exon 9 (706 bp, Reverse 2).

Forward: GTCTCCTAGGAACAATCCAATCA (exon 2a)

Reverse 1: ACTGGCAGATGAGTGTCTCC (3' end of sauropsid-like exon 8)

Reverse 2: CTTGTGGGGCAGATTAGAGG (3' end of mammal-like UTR of exon 9)

RNAs were purified (RNeasy Midi Kit; Qiagen, France), and converted into cDNA (M-MLV Reverse Transcriptase, Invitrogen) using an oligo(dT)18 primer. *AMTN* transcripts were recovered by PCR amplification (annealing temperature: 58°C) using GoTaq DNA polymerase (Promega, France), as previously described [13]. Sequencing was performed by GATC Biotech. We used SeaView 4.5.2 for sequence alignment [14].

For sequence comparison we used the published *AMTN* cDNA of the rodent *Mus musculus* (Genbank accession NM_027793.1), the crocodile *Caiman crocodilus* (KM069444) and the lizard *Anolis carolinensis* (KM069435).

The probe for *in situ* hybridization was synthesized as previously described [4] using the *AMTN* transcript fragment amplified with primer set 2 (706 bp), i.e. possessing a large common sequence of the two transcripts and differing from transcript 1 in the 3' region only (49 bp). Briefly: purified cDNAs were inserted into a vector containing T7 and SP6 promoters for *in vitro* RNA transcription, and transformed into competent *E*. *coli*. Colonies containing the vector and the insert were selected, plasmids purified and linearized by PCR, and the antisense RNA probe was synthesized in the presence of digoxigenin.

### Transcript quantification

Relative abundances of the two transcripts were estimated by remapping the reads on the transcriptome assembly with Bowtie2 [15] and performing RSEM (RNA-Seq by Expectation Maximization) abundance estimation [16]. For each transcript the FPKM (Fragments Per Kilobase Of Exon Per Million Fragments Mapped) values and abondance estimation of variants were computed.

### *In situ* hybridization

The fixed jaws were dehydrated and embedded in paraplast. The sections (8 μm-thick) were deposited on slides, dewaxed in toluene, rehydrated, treated with proteinase K, post-fixed in paraformaldehyde, rinsed and incubated overnight with the digoxigenin-labeled antisense probe in the hybridization buffer (see procedures in [4]). The following day, the slides were washed three times, rinsed in the maleic acid buffer tween, non-specific binding sites blocked, and incubated overnight with the anti-digoxigenin antibody coupled to alkaline phosphatase. Then, the slides were rinsed, the digoxigenin-labeled probes revealed by immunochemistry, mounted and photographed.

## Results

### AMTN gene structure

Screening the assembled jaw transcriptome using the putative *AMTN* sequence of opossum gDNA provided two transcripts with high e-value. They were 1138 bp and 1730 bp long. Alignment with the putative gDNA sequence revealed that these transcripts corresponded to two *AMTN* variants, variant 1 (V1) and variant 2 (V2) respectively, and included the 3' and 5' UTR. The transcripts shared the same sequence, including a 119 bp-long 5' UTR, and differed in their 3' region. The shortest transcript, V1, possessed a short exon 8 and ended with exon 9, encoding the three last residues, the stop codon and a 326 bp-long 3' UTR. The largest transcript, V2, possessed a large exon 8, encoding a RGD motif, the stop codon and a 669 bp-long 3' UTR. A third variant (V3), not found in the transcriptome, was identified when cloning the transcripts for probe synthesis. V3 ended with exon 9 but lacked exon 7, resulting in the absence of the putative phosphorylation site (SxE motif) in the encoded protein. The three cDNA sequences were deposited in GenBank under accession number KP184475, KP184476 and KP184477, respectively.

**Fig 1:**
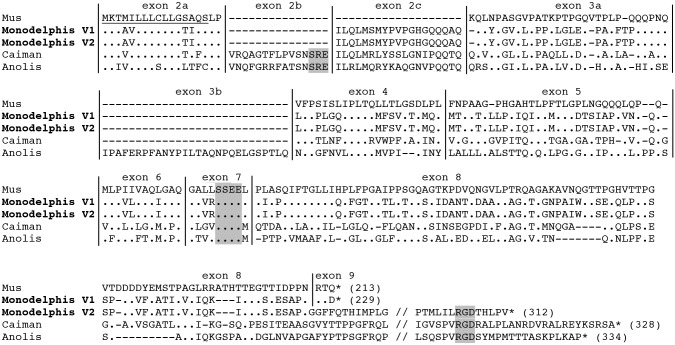
Amelotin sequence alignment. The two main *AMTN* variants (V1, V2) of *Monodelphis domestica* are aligned with rodent (*Mus musculus*), crocodile (*Caiman crocodilus*) and lizard (*Anolis carolinensis*) sequences. Fonctionally important motifs (SxE motifs encoded by exon 2b and exon 7, and the RGD motif encoded at the end of exon 8) are highlighted in grey. Signal peptide underlined; sequence length indicated in brackets; exon limits indicated by vertical lines; (.): residue identical to *M*. *musculus AMTN* residue; (-): indel; *: stop codon. (//): for convenience of presentation the sequences were shortened.

The translated amino acid sequences of V1 and V2 were aligned with the mouse, crocodile and lizard AMTN sequences (Fig 1).

The three variants possessed the exon 2c previously identified as putatively functional in opossum gDNA, but lacked exon 2b, which is present in non-mammalian tetrapods and encoded an SxE (SRE) motif. The putative phosphorylation site encoded by exon 7, and conserved in all AMTN sequences analyzed so far, is found in V1 and V2, but not in V3. The RGD motif, encoded by all non-mammalian *AMTNs*, was only encoded by the 3' region of V2.

### Relative abundance of AMTN mRNA variants

The FPKM values (number of normalized reads) for V1 and V2 (V3 not identified in the transcriptome) were 6.39 and 2.64, respectively. FPKM%, i.e. the percentage of the two *AMTN* transcripts related to all transcripts, were 0.00115 and 0.00047, respectively, which means that V1 represented 70.79% *vs* 29.21% for V2 of all *AMTN* transcripts in the sequenced jaw transcriptome.

### Expression of AMTN during amelogenesis

**Fig 2:**
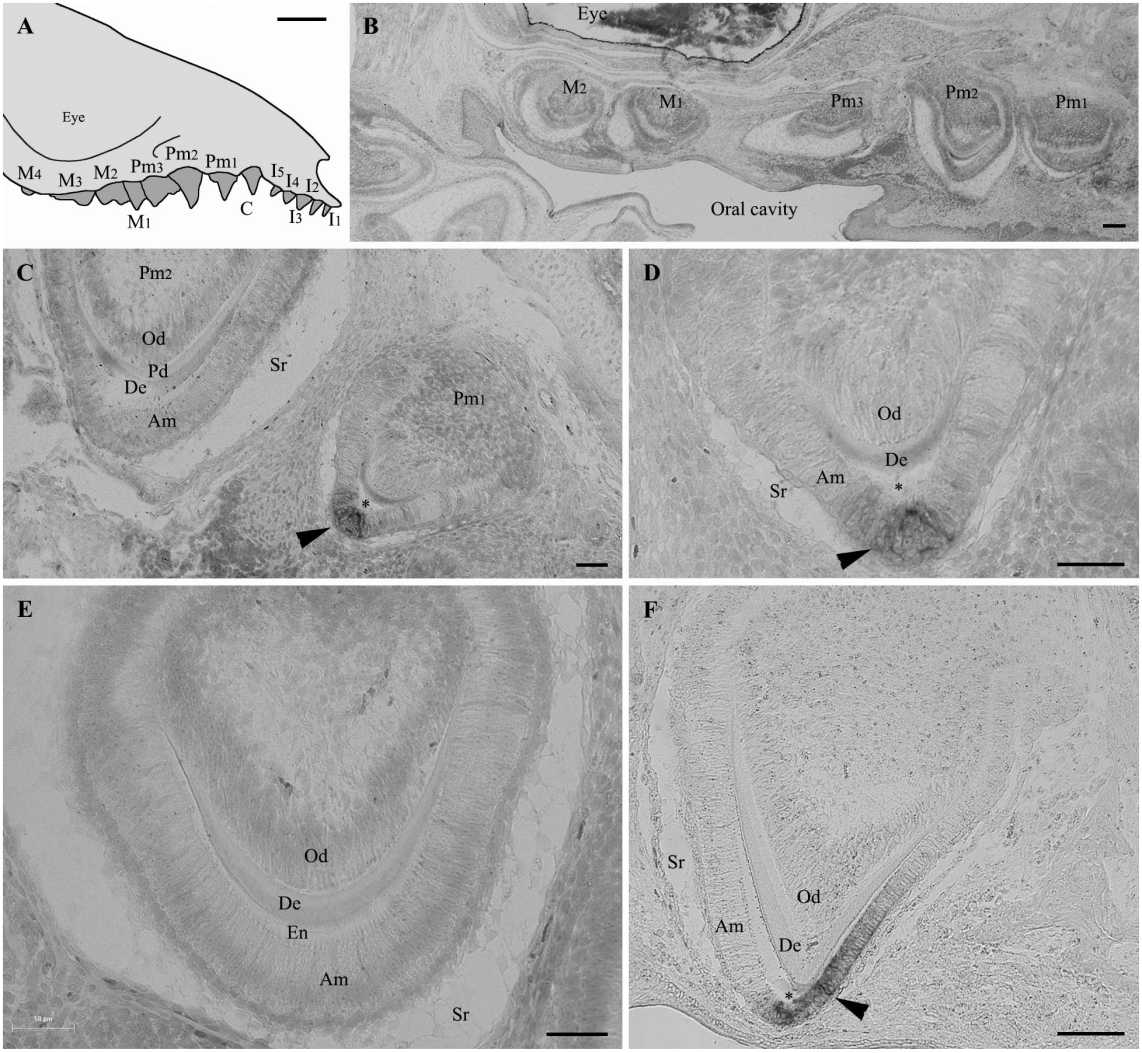
Amelotin gene expression during amelogenesis in *Monodelphis domestica* teeth. (A) Schematical drawing of the upper jaw of a *Monodelphis domestica* neonate (lateral view). (B-F) *In situ* hybridization of sagittal tooth sections in the upper jaw of a 16 days neonate using *AMTN* probe. (B) Low magnification of a section showing five developing teeth. No labelling of *AMTN* transcripts are observed although the teeth are already formed and show well-differentiated ameloblasts. Enamel matrix is present but not yet undergoing maturation process, at least in this section level. (C) Section of premolars 2 (left) and 1 (right). In the less developed premolar 2 (secretory stage), the ameloblasts, which are well-differentiated, display Tomes' processes and are facing recently formed enamel matrix, are not labelled. In contrast, in premolar 1, labelled *AMTN* transcripts (arrowhead) are observed in a few ameloblasts located at the tooth tip and facing the mineralized enamel (asterisk). (D) Higher magnification of Pm1 in (C). Mature enamel (asterisk) facing labelled ameloblasts (arrowhead) was removed during the demineralization process. (E) Similar stage as Pm2 in (C). The secretory-stage ameloblasts display Tomes' processes but do not express *AMTN*. Immature enamel matrix is present between the ameloblasts and the dentin layer. (F) In the upper region of premolar 1 *AMTN* expression is only located in the short, maturation-stage amelobasts facing the well-mineralized enamel (asterisk). The ameloblasts facing the immature enamel matrix (on the left) are not labelled. Am: ameloblasts; De: dentin; En: enamel matrix; Od: odontoblasts; Pd: predentin; Sr: stellate reticulum; *: enamel space. C: canine; I: incisor; M: molar; Pm: premolar. Scale bars: A = 2 mm; B, F = 100 μm; C, D, E = 50 μm.

Several teeth were forming in the jaws of the opossum neonate we used (Fig 2). Serial, sagittal sections of demineralized upper and lower jaws revealed various stages of amelogenesis depending on the section level. Most teeth were well developed and exhibited features indicating well-differentiated ameloblasts (Fig 2B–2F). Tooth sections revealed a well-developed dental organ with differentiated, polarized odontoblasts facing the predentin matrix, and a well-developed enamel organ, with prominent stellate reticulum and differentiated, polarized ameloblasts. *In situ* hybridization did not identify *AMTN* transcripts in the differentiated ameloblasts facing recently deposited enamel matrix (Fig 2B, 2C and 2E). In contrast, in sections through tooth regions displaying a well-mineralized or mature enamel, *AMTN* mRNA were clearly identified in the maturation-stage ameloblasts (Fig 2C, 2D and 2F). Well-mineralized or mature enamel was easily recognizable as an empty space devoid of matrix resulting from the demineralization process. Careful examination of all serial sections of upper and lower jaws did not reveal other labelled cells, neither odontoblasts nor cells located along the tooth base. These findings indicate that *AMTN* transcription is activated late in the ameloblasts during opossum amelogenesis, from the transition stage (advanced mineralization) onwards.

## Discussion

### AMTN gene structure changed during mammalian evolution

Previous analyses of gDNA sequences led to the hypotheses that either all mammalian *AMTN* sequences, including that of the opossum, had the same structure as described in rodents, i.e. the variant 1 sequence [9], or the gene structure in opossum differed in possessing a large exon 8, i.e. variant 2 sequence, as in non-mammalian tetrapods [2], a structure also predicted in GenBank (ENSMODT00000015781). The discovery of V1 and V2 transcripts in the opossum jaw transcriptome indicates that both hypotheses were not entirely correct and brings further clues to AMTN evolution. We demonstrate here that the mutation creating an intra-exonic splice site in *AMTN* exon 8 and resulting to V1 occurred before marsupial and placental lineages diverged (more than 180 million years ago [17]). Moreover, the presence of V2 in the opossum transcriptome indicates that the splicing was primarily alternative. The donor splice site resulting in skipping the 3' region of exon 8 (and hence the stop codon) took place using a GTG sequence, which changed into the more common GTA sequence in a placental ancestor [4]. The lack of V2 in placental *AMTN* indicates that the alternative splicing is no longer present [9]. We believe that the intra-exonic splicing was favored when GTG changed into GTA in an ancestral placental, and that selective pressure on V2 was relaxed, probably because the encoded region, including the RGD motif was less useful.

The relative expression level of V1 and V2 in opossum jaw transcriptome provided by the estimated FPKM values is not accurate given the lack of replicates but predominance of V1 (70.8 vs 29.2%) indicates that this transcript is being selected in the marsupial lineage, probably as having the major function. The gDNA sequence of platypus *Ornithorhynchus anatinus AMTN* both revealed the presence of a similar, putative intraexonic splice site in exon 8 (potentially V1 transcript) and the lack of encoded RGD motif if a putative V2 was translated [9]. This strongly suggests that the intraexonic splice site in exon 8 could have occurred earlier in mammalian evolution.

The *AMTN* transcript lacking exon 7, V3, was only found when cloning PCR products and was not identified in the transcriptome assembly. However, PCR and transcriptome sequencing were performed from the same RNA extraction. This means that V3 transcripts were present but probably at a too low amount to be assembled after transcriptome sequencing. V3 sequence was obtained at random when cloning. It is worth noting that such variant is known in rat *AMTN* [5], which indicates that such alternative splicing have occurred in various mammalian lineages.

The presence of exon 2c in the three variants confirms previous predictions by screening the opossum gDNA and comparing tetrapod sequences [2,4]. Exon 2c was present in the last common tetrapod ancestor, conserved in early mammals, including monotremes and marsupials, and then lost in placentals, which indicates that the encoded region was not important for the protein function in this lineage.

We confirm the lack of exon 2b in opossum transcripts as previously observed in cDNA sequences of rodent *AMTN* [3,5]. In non-mammalian tetrapods, exon 2b encodes an SxE motif that probably plays an important role as a phosphorylation site. Moreover such motif characterizes many SCPP family members [18]. The loss of this motif in the mammalian lineage is intriguing and could indicate a change in AMTN function during mammalian amelogenesis compared to that of other tetrapods, but this hypothesis remains to be tested.

Opossum AMTN possesses, however, a putative phosphorylation site encoded by exon 7 as described in rodents [3,5]. Recently, Abbarin et al. [19] showed that the synthetic phosphopeptide pSpSEEL was able to promote hydroxyapatite (HA) precipitation and that both Ser phosphorylations are important in the mineralizing ability of AMTN. Conservation of this SxE motif through mammalian evolution while the other SxE was lost suggests different function for these two sites during amelogenesis. Here also further functional studies are needed to understand the respective role of these two sites. The short phosphopeptide pSpSEEL promoted HA precipitation from the solution, while the nonphosphorylated peptide had no effect on mineralization. However, the full-length recombinant human AMTN protein promoted a better mineral precipitation, which suggests that other mineral-interacting sites could exist on the sequence and are required for a fully functional protein [19].

### A late spatio-temporal AMTN expression was defined early in mammalian evolution

We demonstrated here that during opossum amelogenesis *AMTN* expression occurs late, when the enamel matrix is well mineralized. This finding contrasts with the results obtained in lizard and salamander, in which *AMTN* is expressed from early enamel matrix deposition to late maturation stage, and even along the dentin shaft when teeth are well formed [4]. In opossum, the *AMTN* probe never labelled ameloblasts at early stages of enamel matrix deposition, nor the inner dental epithelial cells located along the tooth base after enamel maturation. Such spatiotemporal expression pattern is similar to that described in mice, in which *AMTN* transcripts were identified from transition to late maturation stages [3]. Taken together these data indicate that, in the last common tetrapod ancestor, AMTN was involved in processes more extended spatially and temporally during tooth development than in marsupials and placentals, with expression patterns similar to the enamel matrix proteins, AMEL, AMBN and ENAM. AMTN expression was restricted early in mammalian evolution (at least at least 180 Ma), playing only a role during late stage of amelogenesis.

### Both gene organization and expression support structure-function relationships

By demonstrating an ancient origin of the current *AMTN* structure and expression pattern in mammalian evolution, our results reinforce our previous hypothesis of a relation between gene (and protein) structure and expression pattern [4]. Without elaborating exagerated speculations it is clear that when AMTN possesses the two SxE (encoded by exons 2b and 7) and the RGD (encoded by the 5' region of exon 8) motifs as in lizards and salamanders, the expression pattern is spatially and temporally extended, and that these features were the ancestral condition in tetrapods. In contrast, when AMTN is lacking the first SxE and the RGD motif, the expression pattern is restricted to the late stages of enamel formation, a feature that could be related to the loss of the two motifs. Further experiments should be designed to reveal the function of these two motifs during amelogenesis in non-mammals and the reasons why they were lost in an ancestral mammal. In the same research field, in our laboratory we are currently performing experiments aiming to check whether the expression patterns of EMPs (AMEL, AMBN and ENAM) are similar or different in rodents and sauropsids as observed for AMTN.

It remains also to know which changed first in an ancestral mammal, the expression pattern or the gene structure. We believe that, early in synapsid evolution (310–200 Ma), an unknown event may have occurred in the regulatory region of *AMTN*, resulting in its late expression during amelogenesis, i.e. no longer involved as an enamel matrix protein. This drastic change in expression pattern could have led to the modification of the protein functions and some previously important motifs probably became no longer useful. Then, two mutations may have occurred: exon 2b (SxE motif) was lost first then alternative intraexonic splicing in exon 8 led to the disappearance of the encoded RGD motif and a shortest transcript. Alternative transcripts first coexisted as variants, the shortest being progressively selected to finally become the only *AMTN* transcript present in placental mammals.
